# Degradation in landscape matrix has diverse impacts on diversity in protected areas

**DOI:** 10.1371/journal.pone.0184792

**Published:** 2017-09-26

**Authors:** Matti Häkkilä, Eric Le Tortorec, Lluís Brotons, Ari Rajasärkkä, Risto Tornberg, Mikko Mönkkönen

**Affiliations:** 1 Department of Biological and Environmental Science, University of Jyväskylä, Jyväskylä, Finland; 2 Center for Mediterranean Forest Research, Centre Tecnològic Forestal de Catalunya, InForest JRU, Solsona, Spain; 3 Metsähallitus, Parks & Wildlife Finland, Oulu, Finland; 4 University of Oulu, Oulu, Finland; University of Fribourg, SWITZERLAND

## Abstract

**Introduction:**

A main goal of protected areas is to maintain species diversity and the integrity of biological assemblages. Intensifying land use in the matrix surrounding protected areas creates a challenge for biodiversity conservation. Earlier studies have mainly focused on taxonomic diversity within protected areas. However, functional and especially phylogenetic diversities are less studied phenomena, especially with respect to the impacts of the matrix that surrounds protected areas. Phylogenetic diversity refers to the range of evolutionary lineages, the maintenance of which ensures that future evolutionary potential is safeguarded. Functional diversity refers to the range of ecological roles that members of a community perform. For ecosystem functioning and long-term resilience, they are at least as important as taxonomic diversity.

**Aim:**

We studied how the characteristics of protected areas and land use intensity in the surrounding matrix affect the diversity of bird communities in protected boreal forests. We used line-transect count and land-cover data from 91 forest reserves in Northern Finland, and land-cover data from buffer zones surrounding these reserves. We studied if habitat diversity and productivity inside protected areas, and intensity of forest management in the matrix have consistent effects on taxonomic, functional and phylogenetic diversities, and community specialization.

**Results:**

We found that habitat diversity and productivity inside protected areas have strong effects on all diversity metrics, but matrix effects were inconsistent. The proportion of old forest in the matrix, reflecting low intensity forest management, had positive effects on community specialization. Interestingly, functional diversity increased with increasing logging intensity in the matrix.

**Conclusions:**

Our results indicate that boreal forest reserves are not able to maintain their species composition and abundances if embedded in a severely degraded matrix. Our study also highlights the importance of focusing on different aspects of biodiversity.

## Introduction

Human activities are a major threat to all aspects of biodiversity. A cocktail of climate change and destruction of habitats [1] mixed together with pollution and invasive species are decreasing biological diversity throughout the world [2]. The remaining fragments of native habitats are affected as a consequence of hindered migration and isolated populations [3], [4], [5].

Despite the multi-faceted nature of biodiversity [6], community ecologists and conservation biologists have only recently considered aspects of biodiversity other than taxonomic diversity. Taxonomic diversity is the most commonly quantified component of diversity, accounting for species richness and abundances. Phylogenetic diversity reflects evolutionary history of the present taxa [7], and measuring it has been proposed as a way to acknowledge the role of species interactions in biotic assemblages [8]. High levels of phylogenetic diversity may enhance the resilience of communities to changing environmental conditions [9]. Functional diversity describes the range of ecological roles that organisms perform, as well as how they use their habitat and resources [10]. For the productivity of ecosystems, functional diversity is of high importance [11]. Taxonomic, phylogenetic and functional diversity in communities tend to be positively correlated across spatial scales [12], but they reflect complementary components of biodiversity. Two communities may possess equal levels of taxonomic diversity, yet be composed of species with different phylogenetic histories or set of functional traits.

Specialist species, by definition, effectively utilize a narrow range of available resources, and communities containing many specialist species may therefore be more efficient in transforming available resources into offspring than communities with predominance of generalists [12]. Unlike generalists, specialists often have specific functional traits; losing them may result in decreased functional diversity, which is an aspect of biotic homogenization [13]. Nevertheless, functional diversity may remain unchanged when specialist species are lost if new generalist species occupy the empty niches. Thus, all ecosystem functions are retained but overall effectiveness of resource use may be considerably reduced. Therefore, quantifying changes in the level of specialization may be a useful additional indicator of important changes in communities.

Landscapes have traditionally been considered as a network of habitat patches in a non-habitable matrix [14]. However, more recent research has shown that the matrix surrounding patches can affect habitat quality [15], dispersal ability of species and population persistence [16], [17] and thus colonization-extinction dynamics [18]. Individuals moving between patches may use alternative resources outside the fragment they occupy [19]. Protected areas are often located in landscapes where land use intensity around them is high and matrix habitats are of poor quality compared to natural habitat. In forested landscapes, for example, protected old-growth forests are often surrounded by clear-cuts or young forests [20], [21]. Protected boreal forest areas embedded in a matrix of young regeneration forests foster less species-rich communities with higher total bird density than areas situated in continuous, old forest dominated landscapes [21]. This supports the idea that the matrix provides additional resources even for patch-dependent species [22], while only a limited set of species are able to utilize resources in the matrix. However, little is known about the matrix effects on the capacity of protected areas to maintain functional and phylogenetic diversity or on community specialization in protected areas. Further, characteristics of protected areas that promote specialized species and their functions have hardly gained any interest (but see [23]). It has been proposed that larger protected areas are needed for the persistence of populations [24] and the integrity of communities [21]. The relationship between functional and phylogenetic diversity, community specialization and habitat area or productivity has remained unclear.

In this research, we studied how characteristics of protected areas and the surrounding matrix impact the community composition of forest birds within the protected areas. Increasing human land-use intensity has negative effects on diversity [25], so we hypothesize that forest bird communities will be negatively affected by intensive forestry in the matrix. Moreover, we hypothesise that matrix effects will be particularly strong in small protected areas, whereas large areas should be better buffered against matrix effects, e.g. due to their lower edge-to-area ratio. Total forest area, productivity and habitat diversity all have positive effects on species richness [26]. Therefore, we expect taxonomic diversity to be the highest in large protected areas with high habitat heterogeneity and high productivity inside the areas and that other diversity metrics will show similar responses. Correlations between different diversity measures are often positive, but there are also areas of clear incongruence [14]. Therefore, we also studied if there are correlations between different diversity measures.

## Material and methods

### Study area

**Fig 1:**
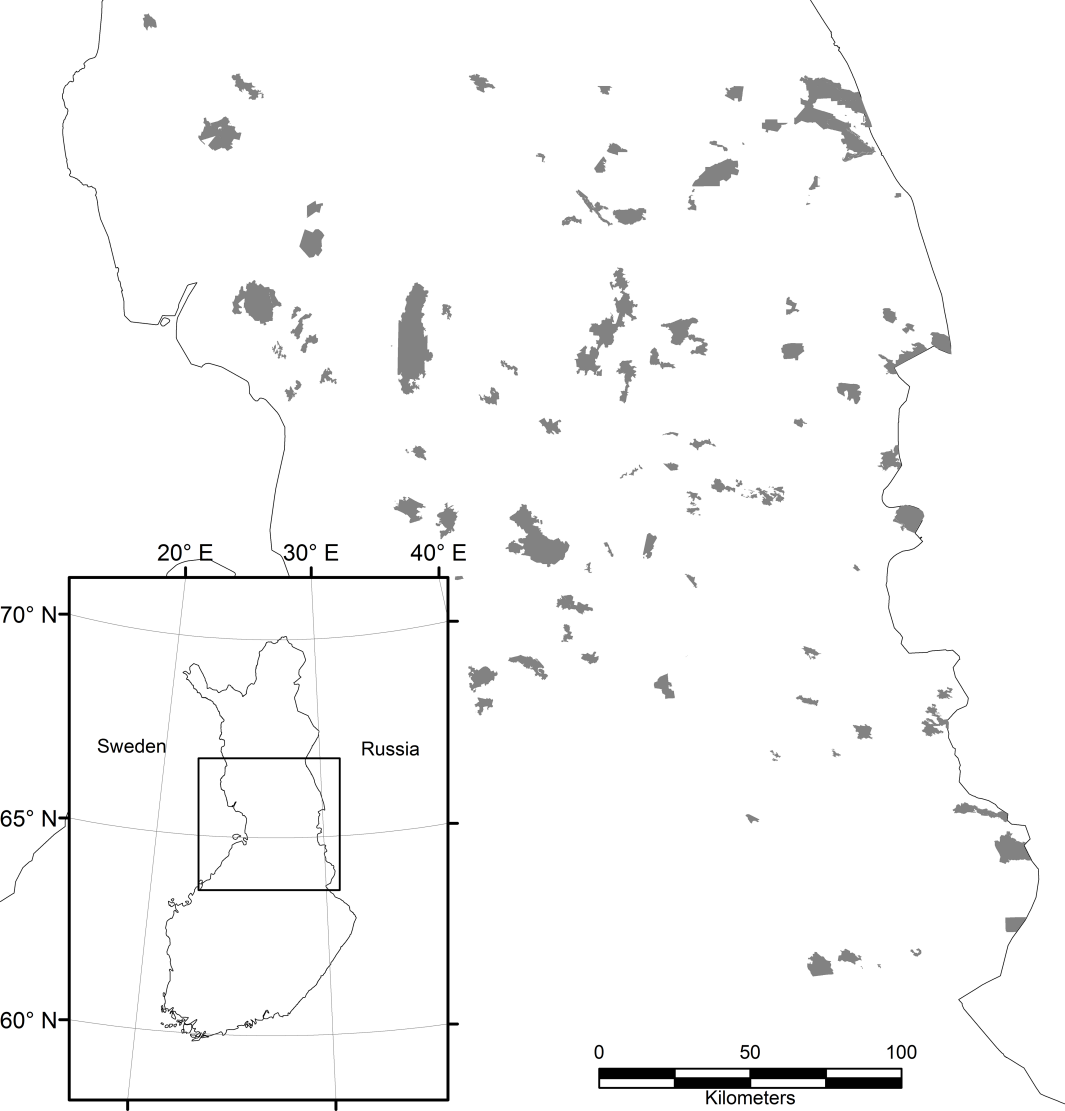
Map of the study area and the location of the protected areas in Northern Finland.

The study area is in the boreal zone in sparsely populated Northern Finland (Fig 1) where forests are mainly dominated by coniferous forests. Open bogs, mires, small lakes and ponds are characteristic of the landscape. Most of the forests are in active commercial use and, in the Kainuu area where most of the studied forest reserves are situated, only 5,2% of forest area is protected from logging [27]. This study focuses on 91 protected areas with a total surface of approximately 3100 km^2^. The areas differ in size, from ca. 150 to 28000 ha (mean area = 3400 ha, S.D. = 4676 ha).

### Bird data

Bird species abundance was measured using the Finnish line transect census method [28] by Metsähallitus Parks & Wildlife Finland. Finnish line transect count is based on observational data with minimum disturbance to animals. It can be accomplished under Finnish Every man’s right, which allows free access without separate permit to all forests, protected areas and National parks included. The line transect census method is a one-visit census and is suitable for counting birds over large areas [29]. The survey is completed early in the breeding season and all observations of breeding pairs in 5 to 6 km transects on average are counted. See S1 Text for further information.

We focused on forest species and of all the 129 bird species observed, we retained 69 species that use forest as their main breeding habitat [30] (S2 Text). The bird censuses were conducted between 1988 and 1999 for a total of 3323 km of transects. On average, 1 km of transect per km^2^ of land within the protected areas was surveyed. Transect length correlated negatively with land area, suggesting that small areas were surveyed with higher per-unit-area effort. In large areas, all the line transect counts could not be conducted during the same breeding season. We combined data across years following Brotons et al. [31] who found that species richness and abundance of forest species in these data did not differ among years.

### Diversity measures

We first calculated species richness in each site. Further, for each of the protected areas, we calculated taxonomic, functional, and phylogenetic α-diversities based on Rao’s quadratic entropy [32] using R functions developed by De Bello et al. [33], as well as the Community Specialization Index [34]. The α-diversity of a community represents the expected dissimilarity between two randomly selected individuals, taking the relative abundance of each species into account. For taxonomic diversity, the dissimilarity has a value of 1 if two individuals represent two different species and 0 otherwise. For functional and phylogenetic diversity measures, dissimilarity takes into account functional or phylogenetic distances among species [35], [33] (see below). For species level taxonomic diversity, α-Rao equals the Simpson diversity index [35], [36]. Thus, a community with few species and one dominant species will get a low value.

Functional diversity is often measured by using ecological traits [37], but in this study we are proposing a method based on morphological traits. The functionality of a species refers to the way it uses the habitat for foraging, nesting, avoiding predators, etc. [38], which is largely reflected in the morphology of a species. We measured morphological traits (wing, tail, tarsus and bill length, bill width and bill height) from at least five individuals per species from permanent museum samples available for scientific use at the Museum of Natural History in Helsinki, Finland, and the Museum of Natural history in Oulu, Finland. Body mass was available from museum databases (weighed when individuals were collected). In the few cases where specimens originating from Finland were not available, we chose the ones collected as close to the study area as possible. As all of these measures mainly are correlated with the size of the bird, we transformed original morphological variables into indices that link morphology with ecological functions of the species. First, we used body mass as an indicator of overall body size. Body size is an important driver of both habitat use and diet, and two species with different body size but equal shape tend to use different size food items and different habitats. Second, to describe functions related to the type of food used, we computed the ratio bill length/(bill width + bill height). Species with long bills relative to bill width and height tend to be more insectivorous than short-billed species [39]. Third, we calculated three additional ratios (wing length/(∛body mass), tarsus length/(∛body mass) and tail length/(∛body mass)) to represent differences in locomotion and habitat use. The lengths were divided by the cubic root of body mass to scale these one-dimensional variables by a one-dimensional measure of body size. These five uncorrelated (see S4 Text) morphological variables were then used to calculate pairwise Euclidean distances in a morphological hyperspace among individuals in a community. This distance, scaled between 0 and 1, was then used as a measure of morphological dissimilarity when calculating functional diversity.

To study phylogenetic diversity in our study area a set of 1000 randomly chosen phylogenetic trees of the study species was chosen from a phylogenetic tree database of 9993 bird species that is available on birdtree.org [40],[41]. Using a large sample is better than using few trees since there can be some uncertainty in phylogeny reconstruction. Of the 1000 trees, a single, unrooted, 50% majority rule (extended) consensus tree was constructed using the Consense function (phylip package v.3.695). In the extended majority rule, any set of species that appears in more than 50% of the trees is included. To calculate phylogenetic diversity, we used the distance between the two end nodes (i.e. species) in the consensus tree, scaled between 0 and 1, as a measure of phylogenetic dissimilarity.

The species specialization index, SSI, [32] is a simple and sensitive tool for studying homogenization at the community level. SSI is the coefficient of variation (standard deviation/average) of species densities among habitat classes. The larger the SSI value, the more specialized the species is with respect to habitats. We used independent Finnish national level data originating from the common bird monitoring project (CBMP) point count censuses, conducted between 1984 and 2011 [42], to calculate the SSI for each species. In the CBMP, each station is located in a uniform habitat within at least 50 m to the station. Habitat type is classified into 17 classes (S9 Text). For each habitat class we derived average density for the 69 species, and calculated sample-size bias corrected species specialization index, SSIc [32]. Finally, we calculated a weighted average SSIc, which takes species density into account, of species present in a given site, i.e. the community specialization index, CSI [43].

### Landscape analysis

The land-use and cover data originated from the 8th National Forest Inventory of Finland and were collected between 1986 and 1994 [44]. In these data, forests were classified by timber volume and dominant tree species into nine cover types (see [31]; Table 1). From this classification, we calculated variables describing the habitat composition within the protected areas and in the surrounding landscape. We used the sum of spruce-deciduous cover types (habitat classes 3 and 6; Table 1) to describe productivity because, in our study area, spruce-deciduous forests only grow on fertile soil while less fertile sand and peat soils are usually pine-dominated. Forests with more timber than 100m^3^/ha can be considered mature old forests in northern Finnish conditions [45]. Consequently, we used the sum of the three cover types with more than 100m^3^/ha (habitat classes 1–3; Table 1) to describe the proportion of old forest, even though we did not directly measure forest age. We estimated habitat diversity within reserves using Shannon’s diversity index from the proportions of the nine cover types in each area.

**Table 1:** Average, minimum, and maximum percentages of the nine cover types inside the protected areas and in the matrix, and total area and habitat diversity inside the areas (HDIV_In).

Class Number		Inside			Matrix		
	Habitat class	$\bar{x}$	min	max	$\bar{x}$	min	max
1	Pine-spruce >100 m^3^/ha %	16.9	0.8	56.2	7.2	0.9	25.1
2	Pine >100 m^3^/ha %	3.3	0.1	19.0	2.6	0.3	8.6
3	Spruce-Deciduous >100 m^3^/ha %	6.8	0.2	38.6	3.2	0.5	10.0
4	Spruce 25–100 m^3^/ha %	12.4	1.4	34.6	9.9	2.5	21.2
5	Pine 25–100 m^3^/ha %	10.9	0.4	27.3	13.1	3.9	25.0
6	Spruce-Deciduous 25–100 m^3^/ha %	10.7	1.9	45.0	14.4	5.4	33.9
7	Pine bogs %	20.0	0.3	50.9	18.6	7.3	33.2
8	Shrubs <25 m^3^/ha %	5.7	0.7	17.9	15.1	5.4	32.9
9	Other open areas %	13.3	0	35.9	15.8	7.1	26.4
	Total area (ha)	3406	141	27884			
	HDIV_In	1.85	1.47	2.12			

The forested landscape around the reserves (the matrix) was characterized from a 5 km buffer around the outer border of each reserve. This radius was selected to make sure that the matrix could have impact on birds with large home ranges. Since clear cutting, which results in an even-age structure of trees within a stand, is the most common way to regenerate forests in the region, timber volume was considered the best estimate of forest age and forestry intensity.

### Statistical analysis

We constructed linear models with the R (version 3.4.0) to analyze the impacts that the characteristics of protected areas and their surrounding landscape matrix have on each diversity metric of the bird communities in the protected areas. Species richness, which is a count variable, was analysed with generalized linear model using a Poisson distribution with a log link function. We used a two- step modeling approach, where the most important variables inside the protected areas were first selected, after which these variables were kept in the model and the most important variables in the surrounding matrix were then selected. In all models, we included N-coordinate as a covariate to control for the well-known decline in diversity with increasing latitude. In the first step, we included and selected the most important variables describing landscape structure within protected areas, assuming that avian community composition is primarily affected by the characteristics of the area itself. The selected variables were the log-transformed total forest area (TFA; the summed area of habitat classes 1–6 in Table 1), the percentage of both young and old spruce-deciduous forests (PROD_In; percentage sum of habitat classes 3 and 6) to describe productivity, and habitat diversity (HDIV_In). In the second step, we selected the most important variables describing landscape structure in the matrix surrounding the protected areas, in addition to keeping the previously selected variables describing the protected areas. These variables were the percentage of old forest (OF_Matrix; Habitat classes 1–3), and the percentage of shrub (SHRUB_Matrix; habitat class 8). The percentage of old forest described long-term logging history in the matrix surrounding the protected areas: the more old forest in the matrix, the lower the overall logging intensity in the past. High values in the percentage of shrub in the matrix indicate high recent logging activity. To assess if the responses were different in large and small protected areas, we also included the interactions between total forest area and the percentage of old forest in the matrix, as well as total forest area and the percentage of shrub in the matrix.

We used Akaike Information Criteria with small sample size correction (AICc) to select the variables to retain at each step, and included only models that were inside 6 AIC units, which gives a 95% chance of retaining the most parsimonious model [46]. According to nesting rule [46], we discarded all models for which a nested version had a lower AICc value. In cases where we had two or more alternative models, we used model averaging to create one best model [47]. Because variables describing the composition of the matrix were entered into the models after within-area variables, they describe the additional effects of the surrounding matrix. All variables were entered as fixed effects, and correlations among the landscape variables were not particularly strong (S3 Text).

## Results

**Fig 2:**
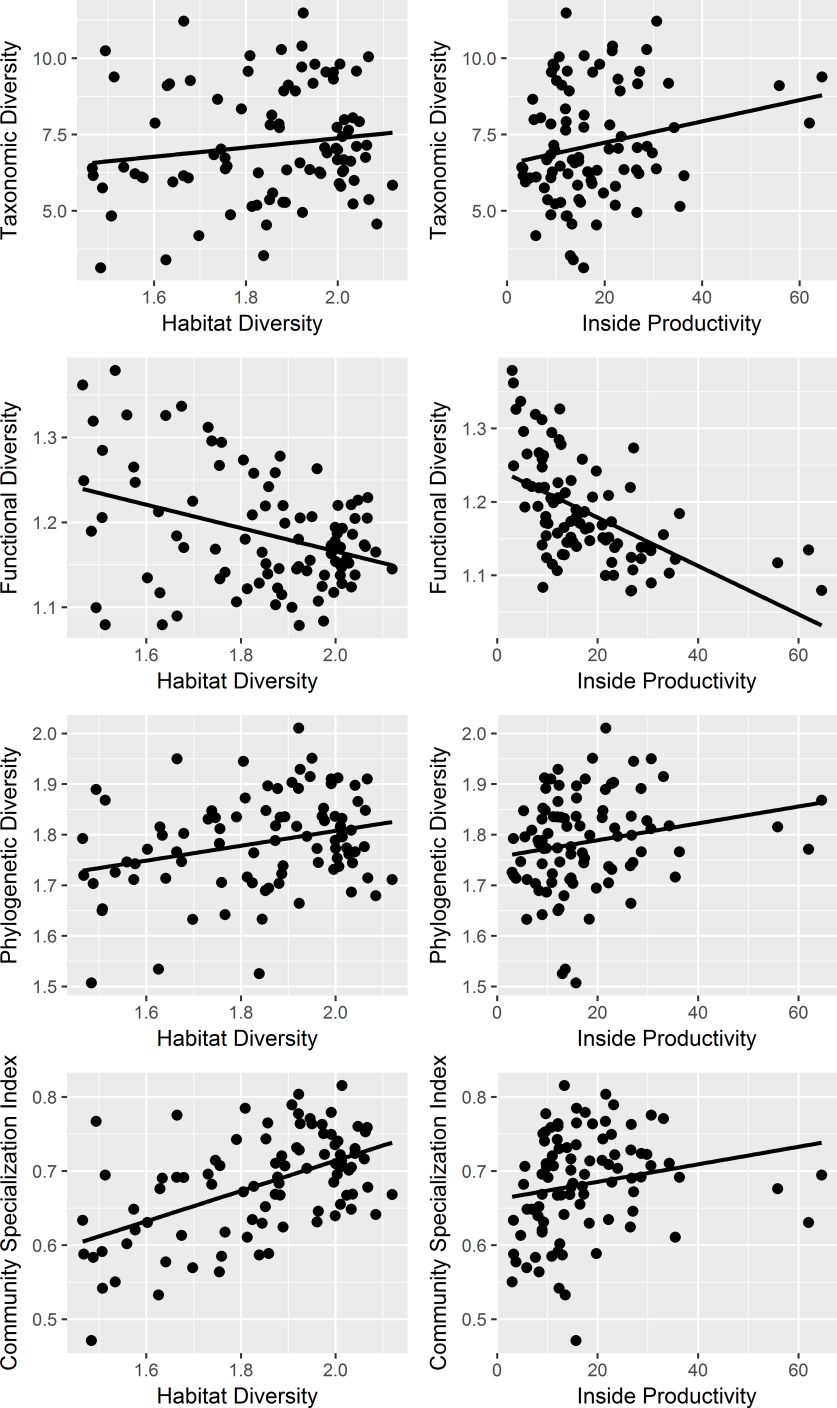
The effects of inside habitat diversity and inside productivity on different diversity metrics.

We found that total forest area and productivity inside the protected areas had positive effects on species richness (Table 2, Fig 2). No matrix effects on species richness were found. The percentage of spruce-deciduous forest (PROD_In) had a strong positive effect on taxonomic diversity, with an increase of 20% along the entire productivity gradient (Table 2, Fig 2). There was also a strong positive impact of habitat diversity (HDIV_In) on taxonomic diversity, with an increase of 30% along the entire gradient of increasing habitat diversity (Table 2, Fig 2). We found no matrix effects on taxonomic diversity. The results for phylogenetic diversity showed very similar patterns as for taxonomic diversity (Table 2, Fig 2).

**Table 2:** Results of two-step regression models best explaining the diversity of Finnish protected forests. Empty cells indicate variables that were not included in the models. For further information about the models, see S6 Text.

		Species richness			Taxonomic Diversity			Functional Diversity			Phylogenetic Diversity			Community Specialization Index		
		Estimate	Std.Error	p	Estimate	Std.Error	p	Estimate	Std.Error	p	Estimate	Std.Error	p	Estimate	Std.Error	p
	(Intercept)	11.17	1.84	<0.000***	106.79	15.24	<0.000***	2.56	0.61	<0.000***	5.203	0.846	<0.000***	0.96	0.67	0.157
Inside	N-coordinate	-0.001	0.04	<0.000***	-0.015	0.0021	<0.000***	-1.53E-04	8.26E-05	0.071	-0.0005	0.0001	<0.000***	-0.0001	0.0001	0.328
	Total Forest Area	0.486	0.039	<0.000*				0.07	0.04	0.046*						
	Productivity (PROD_In)	0.007	0.002	<0.000***	0.069	0.014	<0.000***	-2.70E-03	5.58E-04	<0.000***	0.003**	0.0008	0.0003***	0.0002	0.0006	0.007**
	Habitat Diversity (HDIV_In)				2.73	0.88	0.0025**	-0.090	0.38	0.0266*	0.193	0.049	0.0001***	0.187	0.039	<0.000***
Interaction with Inside forest area and matrix	Forest Area*Shrub in Matrix							-5.092E-03	2.368E-03	0.034*						
Matrix	Old Forest (OF_matrix)							-3.40E-03	1.20E-03	0.005**				0.033	0.0013	0.015*
	Shrub (Shrub_matrixI							-1.41E-03	6.12E-03	0.818						

*p>0.05

** p>0.01

*** p>0.001

**Fig 3:**
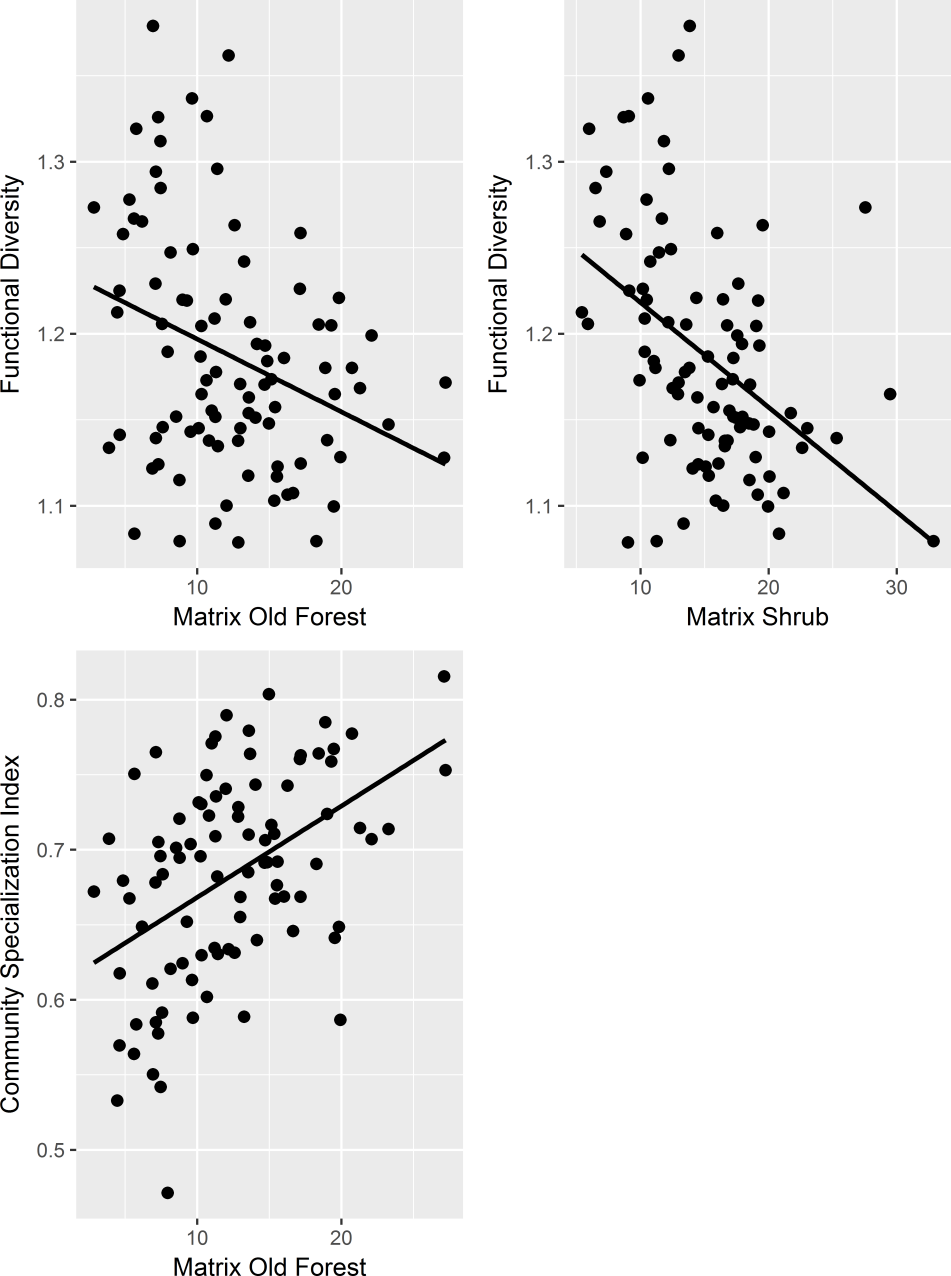
Matrix effects on different diversity metrics.

In contrast to taxonomic and phylogenetic diversity, there was a strong negative influence of the percentage of spruce-deciduous forest within protected areas (PROD_In) on functional diversity (Table 2, Fig 2), with functional diversity monotonically decreasing by approximately 20% along the entire gradient of PROD_In (Fig 2). Also, habitat diversity (HDIV_In) had a negative impact on functional diversity, with a decrease of approximately 10% along the gradient. The effect of total forest area on functional diversity was positive but weak. Of the matrix effects, the percentage of old forest (OF_Matrix) had a negative impact, resulting in a roughly 10% decrease in functional diversity over the gradient of old forest cover (Table 2, Fig 3). The main effect of percentage of shrub was not significant, but the negative interaction between total forest area and percentage of shrub suggested that the effect of recent logging activity was stronger in small protected areas.

Community specialization was positively associated with PROD_In and HDIV_In, showing a roughly 12% increase along the increasing gradients (Table 2, Fig 2). Of the matrix effects, OF_Matrix showed a strong positive impact on community specialization, with a nearly 25% increase along the gradient of increasing old forest (Table 2, Fig 3). Thus, protected areas with high productivity foster more specialized bird communities, especially if they have a high proportion of old forest in the matrix.

Concerning the relationships between the response variables, taxonomic diversity was strongly correlated with phylogenetic diversity (Table 3). There was also a relatively strong correlation between the community specialization index and phylogenetic diversity. Functional diversity was weakly negatively correlated with all other diversity metrics.

**Table 3:** Correlation matrix of different diversity metrics.

	Species Richness	Taxonomic Diversity	Functional Diversity	Phylogenetic Diversity	Community Specialization Index
Species Richness	1				
Taxonomic Diversity	0.417	1			
Functional Diversity	-0.170	-0.186	1		
Phylogenetic Diversity	0.355	0.910	-0.170	1	
Community Specialization Index	0.416	0.683	-0.518	0.789	1

## Discussion

In this study, matrix effects were found for functional diversity, and community specialization, but not for species richness, taxonomic and phylogenic diversity. The amount of old forest in the matrix had a positive influence on community specialization but a negative influence on functional diversity. Inside protected areas, the impacts of productivity and total forest area on species richness were positive, as expected.

The productivity and habitat diversity within protected areas had a strong positive effect on both taxonomic and phylogenetic diversity, as well as on community specialization (Table 2). The pattern supports our hypothesis, as well as earlier literature, that habitat diversity and productivity have a positive effect on species diversity [26]. There were more species in large areas, but larger forest areas did not have increased taxonomic nor phylogenetic diversities. Thus, it seems that the additional species that are occupying large areas are low in abundance and not phylogenetically distinct.

Taxonomic diversity was strongly positively correlated with phylogenetic diversity (Table 3). Thus, increasing taxonomic diversity was associated with an increased phylogenetic distance among species, i.e. with the addition of phylogenetically unique species. Community specialization was relatively strongly correlated with taxonomic and phylogenetic diversity (Table 3). This suggests that increasing taxonomic diversity is associated with an increasing number of specialist species in a community, and that specialist species tend to represent distinct phyletic lineages. In earlier research, overall positive covariation has been found between different facets of biodiversity, but some incongruence has also been found [12]. For example, in their study on spatial distribution and abundance of birds in France, Devictor et al. [12] found that regions with high versus low functional diversity do not necessarily spatially overlap with high vs low taxonomic or phylogenetic diversity. Even though in the French bird data different facets of biodiversity were positively associated, variation in taxonomic diversity explained only a very small proportion of variation (6%) in functional diversity. We found that functional diversity was negatively correlated, although weakly, with all other diversity metrics (Table 3), suggesting that different aspects of species diversity may respond in dissimilar fashion to environmental variation and change.

Functional diversity showed the opposite responses to other diversity measures, with negative impacts of productivity and habitat diversity inside protected areas (Table 2, Fig 2). We suggest that the mechanism for this phenomenon could be explained by species interactions and the properties of Rao’s quadratic entropy in measuring functional diversity. Functional diversity is influenced both by species abundance-based diversity and by functional differences among species [48] [49]. Thus, if one species from a functionally similar group of species becomes less abundant or goes extinct, functional diversity increases. Likewise, the introduction of new species will increase species abundance-based diversity, but it may decrease the average functional dissimilarity among species. On the other hand, the colonization of functionally distinct species will increase average functional dissimilarity between species. A similar effect may stem from an increase in abundance of originally rare but functionally distinct species. The response of functional diversity also depends on the way it has been calculated. Counter to our results, Petchey et al. [50] found that functional diversity increased with increasing species richness. However, unlike in our study, branch lengths of the dendrogram of species (based on functional differences) are summed in their approach: when new species are added into the community the number of branches increases, leading to higher functional diversity.

Our observation that functional diversity decreases with increasing productivity is in line with the Physiological Tolerance Hypothesis [51], postulating that diversity varies according to the tolerances of individual species for different sets of climatic conditions. Accordingly, in less favorable conditions species do not share physiological traits and combinations of traits, and their overlap increases with increasing suitability of ambient conditions. Interestingly, functional diversity was positively influenced by total forest area (Table 2). Because species richness also increased with forest area, it seems that the species that occupy larger areas are also functionally distinct.

The matrix effects that explained functional diversity were the percentage of old forest and, in small areas, shrub in matrix; both had negative effects on functional diversity (Table 2, Fig 3). Thus, functional diversity decreases with increasing recent logging intensity (i.e. shrub) on small areas, but increases with increasing proportion of old forest, i.e. with decreasing overall logging intensity, independent of the size of the area. Thus, large protected areas seem to be better buffered against matrix effects because the effects of recent logging intensity decrease with increasing total forest area. The explanation can be found again in the diversity index, as functional diversity decreases with increasing functional similarity among species. Here, increasing recent logging intensity reduces the average functional distance among species, and thereby results in decreased functional diversity. This may be because of spill-over of generalist species from the matrix. In summary, logging activity in the matrix results in a loss of ecological functions and in biotic homogenization, particularly in small protected areas and in the short-term but not in the long-term.

Community specialization was positively correlated with taxonomic and phylogenetic diversities (Table 3), and was likewise positively associated with habitat diversity and productivity inside protected areas (Table 2, Fig 2). Thus, bird communities in areas with productive forests and a wide variety of habitats have more specialized lineages but, as discussed earlier, less species with similar functions. The positive influence of habitat diversity inside protected areas on community specialization index may first sound counterintuitive because specialist species should benefit from uniform habitats allowing larger population sizes, which decreases the likelihood of stochastic extinctions. We suggest that the effect of habitat diversity on specialist species’ habitat availability is scale-dependent [52]. At small spatial extents, habitat diversity should have a negative effect on specialist species, because for a given area, the amount of area available for individual species decreases with increasing habitat heterogeneity, thereby increasing the likelihood of stochastic extinctions. When total habitat area is large, the negative effect of increasing habitat heterogeneity is balanced by the positive effects of colonization by new specialist species.

The proportion of old forest in the matrix had a positive impact on community specialization (Table 2, Fig 3). This supports our hypothesis that protected areas embedded in an intensively managed matrix foster more homogeneous, less specialized bird communities than those embedded in a less managed matrix. This is caused by the replacement of old forest specialists by generalist species [53]. Our results support the idea that the flow of generalists from the matrix into the protected areas may be an important mechanism of homogenization. However, one could expect this matrix effect to be area-dependent, i.e. small protected areas would be more vulnerable to generalists’ invasion, but we did not find evidence for this with the community specialization index.

## Conclusions

To conclude, our results highlight how different measures of diversity may show different responses to environmental change, which emphasizes the importance of considering multiple aspects when analyzing biodiversity. Our results show that by concentrating only on the conservation of species richness, taxonomic diversity or specialist species, all functions are not necessarily safe-guarded. Even if we manage to maintain ecosystem functions, we may lose unique specialists.

Our results provide insights when establishing new protected areas. If large areas with high species richness are favored, this may not necessarily ensure high taxonomic or phylogenetic diversities, and a focus on species-rich sites may in fact minimize functional diversity. Taxonomic and phylogenetic diversities seem to be secured as long as the area is productive and has high habitat diversity. From a conservation perspective, this is challenging since traditionally protected areas are established in areas where productivity is low and possibilities for exploitation are poor [54]. On the other hand, the response of functional diversity to landscape structure is much more complex than in the case of taxonomic and phylogenetic diversity. For the protection of functional diversity in communities, the matrix is important in addition to the properties of the protected area itself. It seems that uniform areas with low habitat diversity embedded in a matrix with little disturbance have the highest functional diversity. Finally, specialist species benefit from protected areas with high productivity and habitat diversity, and from matrices with a large proportion of old forest. Thus, for specialist species and to prevent biotic homogenization, the matrix quality is of great importance.

## Supporting information

S1 Text Line transect count method. (DOCX)

S2 Text Species list. (DOCX)

S3 Text Correlations between landscape variables. (DOCX)

S4 Text Correlations between morphological indeces. (DOCX)

S5 Text VIF values of model variables. (DOCX)

S6 Text Model rankings. (DOCX)

S7 Text R packages. (DOCX)

S8 Text Phylogenetic tree. (TRE)

S9 Text Habitat categories of Finnish Point Counts. (DOCX)

S1 Table Bird and landscape data. (XLSX)
