# Supplementary material for: Degradation in landscape matrix has diverse impacts on diversity in protected areas
Source: PLoS One. 2017 Sep 26;12(9):e0184792. doi: 10.1371/journal.pone.0184792 (PMC5614538; doi:10.1371/journal.pone.0184792)
Supplement: S1 Text — (DOCX) [file pone.0184792.s001.docx]

Line transect count method

Line transect census method is a one-visit census and it is suitable for counting birds over large areas. The census is completed during early mornings in June when singing activity is highest and the birds are counted along a transect with an average length of 5-6 km. In this method the main belt, which is 50 m wide along the walking transect (25 m on each side of the transect line), and a supplementary belt, which includes all observations beyond the main belt, are separated. In line transect count the study unit is a pair of birds. A singing male, otherwise observed male or female or a group of fledglings are inferred as a pair (Järvinen & Väisänen 1976, Järvinen et al. 1991). From the census data, the Crossbill (Loxia spp) observations were excluded because their nesting season starts already as early as February and thus probably most of the Crossbills observed in the line transect counts were not breeding in the area and therefore did not belong to those communities.
