# Supplementary material for: Degradation in landscape matrix has diverse impacts on diversity in protected areas
Source: PLoS One. 2017 Sep 26;12(9):e0184792. doi: 10.1371/journal.pone.0184792 (PMC5614538; doi:10.1371/journal.pone.0184792)
Supplement: S2 Text — (DOCX) [file pone.0184792.s002.docx]

*Forest bird species used in this study. We did not include species that mainly live on peatlands or wetlands.*

Black Grouse (*Tetrao tetrix*)

Western Capercaillie (*Tetrao urogallus*)

Hazel Grouse (*Bonasa bonasia*)

Merlin (*Falco columbarius*)

Hobby (*Falco subbuteo*)

Honey Buzzard (*Pernis apivorus*)

White-tailed Eagle (*Haliaeetus albicilla*)

Sparrowhawk (*Accipiter nisus*)

Goshawk (*Accipiter gentilis*)

Common Buzzard (*Buteo buteo*)

Golden Eagle (*Aquila chrysaetos*)

Eurasian Woodcock (*Scolopax rusticola*)

Green Sandpiper (*Tringa ochropus*)

Woodpigeon (*Columba palumbus*)

Common Cuckoo (*Cuculus canorus*)

Eagle Owl (*Bubo bubo*)

Ural Owl (*Strix uralensis*)

Great Grey Owl (*Strix nebulosa*)

Hawk Owl (*Surnia ulula*)

Pygmy Owl (*Glaucidium passerinum*)

Tengmalm’s Owl (*Aegolius funereus*)

Northern Wryneck (*Jynx torquilla*)

Lesser Spotted Woodpecker (*Dendrocopos minor*)

Great Spotted Woodpecker (*Dendrocopos major*)

Three-toed Woodpecker (*Picoides tridactylus*)

Black Woodpecker (*Dryocopus martius*)

Eurasian Jay (*Garrulus glandarius*)

Siberian Jay (*Perisoreus infaustus*)

Common Raven (*Corvus corax*)

Carrion Crow (*Corvus corone*)

Bohemian Waxwing (*Bombycilla garrulus*)

Willow Tit (*Parus montanus*)

Siberian Tit (*Parus cinctus*)

Coal Tit (*Parus ater*)

Crested Tit (*Parus cristatus*)

Great Tit (*Parus major*)

Long-tailed Tit (*Aegithalos caudatus*)

Icterine Warbler (*Hippolais icterina*)

Willow Warbler (*Phylloscopus trochilus*)

Common Chiffchaff (*Phylloscopus collybita*)

Wood Warbler (*Phylloscopus sibilatrix*)

Arctic Warbler (*Phylloscopus borealis*)

Greenish Warbler (*Phylloscopus trochiloides*)

Garden Warbler (*Sylvia borin*)

Blackcap (*Sylvia atricapilla*)

Lesser Whitethroat (*Sylvia curruca*)

Winter Wren (*Troglodytes troglodytes*)

Goldcrest (*Regulus regulus*)

Spotted Flycatcher (*Muscicapa striata*)

Pied Flycatcher (*Ficedula hypoleuca*)

Red-breasted Flycatcher (*Ficedula parva*)

European Robin (*Erithacus rubecula*)

Red-flanked Bluetail (*Tarsiger cyanurus*)

Common Redstart (*Phoenicurus phoenicurus*)

Tree Pipit (*Anthus trivialis*)

Eurasian Treecreeper (*Certhia familiaris*)

Eurasian Blackbird (*Turdus merula*)

Fieldfare (*Turdus pilaris*)

Redwing (*Turdus iliacus*)

Song Thrush (*Turdus philomelos*)

Mistle Thrush (*Turdus viscivorus*)

Dunnock (*Prunella modularis*)

Chaffinch (*Fringilla coelebs*)

Brambling (*Fringilla montifringilla*)

Eurasian Siskin (*Carduelis spinus*)

Common Redpoll (*Carduelis flammea*)

Pine Grosbeak (*Pinicola enucleator*)

Eurasian Bullfinch (*Pyrrhula pyrrhula*)

Rustic Bunting (*Emberiza rustica*)
