# Supplementary material for: Degradation in landscape matrix has diverse impacts on diversity in protected areas
Source: PLoS One. 2017 Sep 26;12(9):e0184792. doi: 10.1371/journal.pone.0184792 (PMC5614538; doi:10.1371/journal.pone.0184792)
Supplement: S3 Text — (DOCX) [file pone.0184792.s003.docx]

*Correlations between landscape variables*

|  | N-COORD | HDIV In | TFA | PROD In | SHRUB Matrix | OF Matrix |
| --- | --- | --- | --- | --- | --- | --- |
| N-COORD | 1 |  |  |  |  |  |
| HDIV In | 0.1439 | 1 |  |  |  |  |
| TFA | 0.1070 | 0.2619 | 1 |  |  |  |
| PROD In | 0.3110 | 0.0724 | <0.00 | 1 |  |  |
| SHRUB Matrix | 0.0964 | 0.3323 | <0.00 | 0.3114 | 1 |  |
| OF Matrix | 0.3336 | 0.3653 | <0.00 | 0.0484 | 0.1328 | 1 |
