# Supplementary material for: Degradation in landscape matrix has diverse impacts on diversity in protected areas
Source: PLoS One. 2017 Sep 26;12(9):e0184792. doi: 10.1371/journal.pone.0184792 (PMC5614538; doi:10.1371/journal.pone.0184792)
Supplement: S4 Text — (DOCX) [file pone.0184792.s004.docx]

*Correlations between morphological indexes*

|  | Mass | Bill  function | Wing  function | Tail  function | Tarsus function |
| --- | --- | --- | --- | --- | --- |
| Mass | 1 |  |  |  |  |
| Bill function | 0.2522 | 1 |  |  |  |
| Wing function | 0.3467 | 0.2553 | 1 |  |  |
| Tail function | 0.1406 | 0.2494 | 0.3642 | 1 |  |
| Tarsus function | 0.1761 | 0.2773 | <0.00 | 0.2007 | 1 |
