# Supplementary material for: Degradation in landscape matrix has diverse impacts on diversity in protected areas
Source: PLoS One. 2017 Sep 26;12(9):e0184792. doi: 10.1371/journal.pone.0184792 (PMC5614538; doi:10.1371/journal.pone.0184792)
Supplement: S5 Text — (DOCX) [file pone.0184792.s005.docx]

*VIF-values of model variables*

|  | HDIV | PROD_IN | N-COORD | FOREST_IN | OFBUF05 | LAND805 | FOREST_IN*LAND805 |
| --- | --- | --- | --- | --- | --- | --- | --- |
| Species Richness | 1.728267 | 1.440561 | 1.491451 | 28.890912 |  |  |  |
| Taxonomic Diversity | 1.037241 | 1.124488 | 1.142270 |  |  |  |  |
| Functional Diversity | 1.491296 | 1.428768 | 1.491296 | 24.458515 | 1.526902 | 1.979714 | 24.456249 |
| Phylogenetic Diversity | 1.037241 | 1.124488 | 1.142270 |  |  |  |  |
| CSI | 1.339064 | 1.201342 | 1.470895 |  | 1.495756 |  |  |
