# Supplementary material for: Degradation in landscape matrix has diverse impacts on diversity in protected areas
Source: PLoS One. 2017 Sep 26;12(9):e0184792. doi: 10.1371/journal.pone.0184792 (PMC5614538; doi:10.1371/journal.pone.0184792)
Supplement: S6 Text — (DOCX) [file pone.0184792.s006.docx]

*Model rankings*

1. **Species Richness**

Model selection Inside

Model selection table

(Int) FOR_IN HDI N_KOO PRO_IN df logLik AICc delta weight

6 11.170 0.4860 -0.0014300 0.006803 4 -270.902 550.3 0.00 0.744

8 11.230 0.4811 0.03778 -0.0014460 0.006907 5 -270.857 552.4 2.15 0.254

2 9.154 0.4826 -0.0011340 3 -277.937 562.1 11.88 0.002

4 9.117 0.4879 -0.04016 -0.0011220 4 -277.884 564.2 13.96 0.001

7 12.850 0.59630 -0.0014760 0.007541 4 -336.086 680.6 130.37 0.000

3 10.500 0.51860 -0.0011140 3 -344.718 695.7 145.44 0.000

5 12.180 -0.0012280 0.005825 3 -349.862 706.0 155.73 0.000

1 10.350 -0.0009612 2 -355.587 715.3 165.04 0.000

Model Selection Outside

Model selection table

(Int) FOR_IN LAN N_KOO OFB PRO_IN FOR_IN:LAN FOR_IN:OFB df logLik AICc delta weight

1 11.170 0.4860 -0.001430 0.006803 4 -270.902 550.3 0.00 0.212

3 9.944 0.4739 -0.001263 0.005900 0.006442 5 -269.841 550.4 0.12 0.200

11 9.332 0.5984 -0.001267 0.064190 0.006127 -0.01110 6 -269.023 551.0 0.78 0.144

4 9.979 0.4747 -0.005016 -0.001260 0.006486 0.007047 6 -269.150 551.3 1.03 0.127

2 11.290 0.4876 -0.004146 -0.001441 0.007341 5 -270.420 551.5 1.28 0.112

12 9.358 0.5999 -0.005077 -0.001264 0.065180 0.006717 -0.01117 7 -268.320 552.0 1.72 0.090

8 9.646 0.5449 0.019460 -0.001262 0.006561 0.006867 -0.004895 7 -268.992 553.3 3.06 0.046

6 11.010 0.5496 0.017440 -0.001444 0.007179 -0.004318 6 -270.295 553.6 3.32 0.040

16 9.137 0.6464 0.012930 -0.001265 0.062860 0.006602 -0.003598 -0.01072 8 -268.236 554.2 3.96 0.029

1. **Taxonomic Diversity**

Model Selection Inside

Model selection table

(Int) FOR_IN HDI N_KOO PRO_IN df logLik AICc delta weight

7 106.80 2.734 -0.01458 0.06871 5 -162.072 334.8 0.00 0.735

8 106.80 -0.009975 2.746 -0.01458 0.06872 6 -162.072 337.1 2.29 0.234

5 103.40 -0.01341 0.06313 4 -166.891 342.2 7.40 0.018

6 102.90 0.398000 -0.01361 0.06365 5 -166.107 342.9 8.07 0.013

3 84.80 2.212 -0.01125 4 -172.693 353.9 19.00 0.000

4 84.71 0.034100 2.171 -0.01125 5 -172.689 356.1 21.23 0.000

1 83.51 -0.01051 3 -175.293 356.9 22.01 0.000

2 82.87 0.359200 -0.01067 4 -174.763 358.0 23.14 0.000

Models ranked by AICc(x)

Model Selection outside

Model selection table

(Int) FOR_IN HDI LAN N_KOO OFB PRO_IN FOR_IN:LAN FOR_IN:OFB df logLik AICc delta weight

1 106.80 2.734 -0.01458 0.06871 5 -162.072 334.8 0.00 0.302

3 105.70 2.311 0.04073 -0.01439 0.06222 6 -161.347 335.7 0.84 0.198

5 107.50 2.778 -0.01468 -0.003365 0.06906 6 -162.067 337.1 2.28 0.096

2 106.80 -0.009975 2.746 -0.01458 0.06872 6 -162.072 337.1 2.29 0.096

12 98.12 1.749000 2.158 0.62810 -0.01447 0.05662 -0.1193 8 -159.765 337.3 2.44 0.089

4 105.70 0.026440 2.277 0.04097 -0.01439 0.06215 7 -161.344 338.0 3.19 0.061

7 105.90 2.323 0.04068 -0.01442 -0.000911 0.06232 7 -161.346 338.0 3.19 0.061

6 107.50 -0.007547 2.786 -0.01468 -0.003303 0.06906 7 -162.067 339.5 4.63 0.030

16 97.51 1.752000 2.123 0.62980 -0.01439 0.002710 0.05631 -0.1196 9 -159.762 339.7 4.90 0.026

8 105.90 0.027210 2.291 0.04092 -0.01443 -0.001119 0.06227 8 -161.343 340.4 5.59 0.018

22 108.70 -0.212900 2.846 -0.01471 -0.094710 0.06982 0.01758 8 -162.032 341.8 6.97 0.009

32 98.91 1.516000 2.207 0.63930 -0.01443 -0.115100 0.05731 -0.1217 0.02266 10 -159.701 342.2 7.30 0.008

24 106.70 -0.114500 2.338 0.04048 -0.01445 -0.064040 0.06287 0.01209 9 -161.326 342.9 8.02 0.005

Models ranked by AICc(x)

1. **Functional Diversity**

Model Selection Inside

Model selection table

(Int) FOR_IN HDI N_KOO PRO_IN df logLik AICc delta weight

7 1.806 -0.1504 -3.889e-05 -0.003381 5 142.853 -275.0 0.00 0.757

8 1.801 0.0018230 -0.1526 -3.888e-05 -0.003383 6 142.865 -272.7 2.27 0.243

6 2.019 -0.0208500 -9.269e-05 -0.003101 5 133.332 -256.0 19.04 0.000

5 1.990 -1.032e-04 -0.003074 4 131.793 -255.1 19.88 0.000

3 2.889 -0.1247 -2.025e-04 4 123.861 -239.3 35.74 0.000

4 2.890 -0.0003473 -0.1243 -2.025e-04 5 123.861 -237.0 37.98 0.000

1 2.961 -2.442e-04 3 118.438 -230.6 44.40 0.000

2 2.995 -0.0189600 -2.358e-04 4 119.381 -230.3 44.70 0.000

Models ranked by AICc(x)

Model Selection Outside

Model selection table

(Int) FOR_IN HDI LAN N_KOO OFB PRO_IN FOR_IN:LAN FOR_IN:OFB df logLik AICc delta weight

7 2.618 -0.07806 -0.002635 -1.596e-04 -0.003424 -0.002604 7 149.221 -283.1 0.00 0.285

16 2.285 0.069790 -0.08708 0.020530 -1.583e-04 -0.003289 -0.002842 -0.004700 9 151.505 -282.8 0.30 0.244

32 2.476 0.037780 -0.07562 0.021820 -1.647e-04 -0.019320 -0.002704 -0.004984 0.003082 10 152.571 -282.4 0.70 0.201

8 2.615 0.002025 -0.08046 -0.002617 -1.600e-04 -0.003439 -0.002607 8 149.238 -280.7 2.37 0.087

5 2.516 -0.10750 -1.428e-04 -0.003265 -0.003040 6 146.495 -280.0 3.10 0.060

24 2.796 -0.029010 -0.07026 -0.002713 -1.655e-04 -0.017220 -0.002477 0.002650 9 149.991 -279.8 3.33 0.054

6 2.511 0.004248 -0.11210 -1.438e-04 -0.003300 -0.003042 7 146.566 -277.8 5.31 0.020

12 1.548 0.073180 -0.12970 0.022620 -5.326e-05 -0.003226 -0.005092 8 147.449 -277.1 5.95 0.015

3 1.869 -0.12500 -0.002443 -4.982e-05 -0.002992 6 145.010 -277.0 6.07 0.014

22 2.664 -0.022420 -0.10430 -1.481e-04 -0.015170 -0.002943 0.002282 8 147.094 -276.4 6.66 0.010

1 1.806 -0.15040 -3.889e-05 -0.003381 5 142.853 -275.0 8.09 0.005

4 1.870 -0.000351 -0.12460 -0.002446 -4.984e-05 -0.002991 7 145.010 -274.7 8.42 0.004

2 1.801 0.001823 -0.15260 -3.888e-05 -0.003383 6 142.865 -272.7 10.36 0.002

Models ranked by AICc(x)

1. **Phylogenetic Diversity**

Model Selection Inside

Model selection table

(Int) FOR_IN HDI N_KOO PRO_IN df logLik AICc delta weight

7 5.203 0.1933 -0.0005269 0.002997 5 100.986 -191.3 0.00 0.754

8 5.208 -0.001677 0.1953 -0.0005269 0.002999 6 100.990 -189.0 2.29 0.240

3 4.244 0.1705 -0.0003818 4 94.147 -179.8 11.44 0.002

6 4.929 0.027340 -0.0004580 0.002638 5 94.547 -178.4 12.88 0.001

5 4.967 -0.0004442 0.002603 4 93.413 -178.4 12.91 0.001

4 4.243 0.000247 0.1702 -0.0003818 5 94.147 -177.6 13.68 0.001

1 4.145 -0.0003248 3 88.862 -171.4 19.82 0.000

2 4.099 0.025730 -0.0003363 4 89.769 -171.1 20.19 0.000

Models ranked by AICc(x)

Model Selection Outside

Model selection table

(Int) FOR_IN HDI LAN N_KOO OFB PRO_IN FOR_IN:LAN FOR_IN:OFB df logLik AICc delta weight

1 5.203 0.1933 -0.0005269 0.002997 5 100.986 -191.3 0.00 0.276

3 5.139 0.1672 0.002513 -0.0005156 0.002597 6 101.883 -190.8 0.50 0.215

12 4.725 0.0946000 0.1600 0.034580 -0.0005200 0.002293 -0.006513 8 103.414 -189.1 2.19 0.092

5 5.156 0.1905 -0.0005200 0.0002159 0.002975 6 100.992 -189.0 2.28 0.088

2 5.208 -0.0016770 0.1953 -0.0005269 0.002999 6 100.990 -189.0 2.29 0.088

7 5.058 0.1622 0.002533 -0.0005038 0.0003687 0.002555 7 101.901 -188.5 2.81 0.068

4 5.137 0.0005607 0.1665 0.002518 -0.0005156 0.002595 7 101.883 -188.4 2.85 0.066

16 4.596 0.0952000 0.1525 0.034940 -0.0005015 0.0005770 0.002226 -0.006581 9 103.460 -186.7 4.57 0.028

6 5.158 -0.0018460 0.1925 -0.0005195 0.0002309 0.002975 7 100.997 -186.6 4.62 0.027

8 5.058 0.0003077 0.1618 0.002536 -0.0005039 0.0003663 0.002554 8 101.901 -186.0 5.22 0.020

32 4.796 0.0615600 0.1646 0.036300 -0.0005082 -0.0162700 0.002370 -0.006880 0.003240 10 103.866 -185.0 6.28 0.012

22 5.357 -0.0365800 0.2026 -0.0005251 -0.0152300 0.003104 0.002973 8 101.326 -184.9 6.37 0.011

24 5.238 -0.0306400 0.1720 0.002441 -0.0005094 -0.0133800 0.002684 0.002642 9 102.164 -184.1 7.16 0.008

Models ranked by AICc(x)

1. **Community Specialization Index**

Model Selection Inside

Model selection table

(Int) FOR_IN HDI N_KOO PRO_IN df logLik AICc delta weight

7 1.5800 0.2243 -1.849e-04 0.001829 5 135.569 -260.4 0.00 0.609

8 1.5380 0.01463 0.2068 -1.848e-04 0.001812 6 136.239 -259.5 0.95 0.378

3 0.9942 0.2104 -9.636e-05 4 130.041 -251.6 8.81 0.007

4 0.9551 0.01579 0.1917 -9.714e-05 5 130.734 -250.8 9.67 0.005

6 1.2420 0.04536 -1.119e-04 0.001430 5 121.907 -233.1 27.32 0.000

2 0.7924 0.04448 -4.588e-05 4 119.283 -230.1 30.33 0.000

5 1.3050 -8.899e-05 0.001372 4 116.481 -224.5 35.94 0.000

1 0.8720 -2.609e-05 3 114.326 -222.4 38.06 0.000

Models ranked by AICc(x)

Model Selection Outside

Model selection table

(Int) FOR_IN HDI LAN N_KOO OFB PRO_IN FOR_IN:LAN FOR_IN:OFB df logLik AICc delta weight

5 0.8698 0.1814 -8.098e-05 0.003266 0.001488 6 138.655 -264.3 0.00 0.336

6 0.8567 0.01230 0.1680 -8.411e-05 0.003165 0.001485 7 139.159 -263.0 1.34 0.172

7 0.8257 0.1686 0.0011380 -7.370e-05 0.003334 0.001300 7 139.072 -262.8 1.51 0.158

8 0.8069 0.01337 0.1528 0.0012550 -7.636e-05 0.003232 0.001276 8 139.669 -261.6 2.73 0.086

22 0.8028 0.02172 0.1653 -8.260e-05 0.007361 0.001450 -0.0008067 8 139.215 -260.7 3.64 0.055

1 1.5800 0.2243 -1.849e-04 0.001829 5 135.569 -260.4 3.88 0.048

16 0.6469 0.04621 0.1496 0.0124700 -7.555e-05 0.003305 0.001163 -0.002278 9 140.092 -260.0 4.35 0.038

2 1.5380 0.01463 0.2068 -1.848e-04 0.001812 6 136.239 -259.5 4.83 0.030

24 0.7399 0.02486 0.1491 0.0012910 -7.431e-05 0.008338 0.001228 -0.0009815 9 139.752 -259.3 5.03 0.027

3 1.5550 0.2144 0.0009509 -1.806e-04 0.001677 6 135.841 -258.7 5.63 0.020

4 1.5070 0.01560 0.1943 0.0010940 -1.799e-04 0.001637 7 136.603 -257.9 6.45 0.013

32 0.5981 0.05442 0.1467 0.0121400 -7.393e-05 0.007413 0.001128 -0.002205 -0.0007900 10 140.146 -257.5 6.77 0.011

12 1.3880 0.04280 0.1924 0.0103700 -1.812e-04 0.001550 -0.001884 8 136.874 -256.0 8.32 0.005

Models ranked by AICc(x)
