# Supplementary material for: Degradation in landscape matrix has diverse impacts on diversity in protected areas
Source: PLoS One. 2017 Sep 26;12(9):e0184792. doi: 10.1371/journal.pone.0184792 (PMC5614538; doi:10.1371/journal.pone.0184792)
Supplement: S7 Text — (DOCX) [file pone.0184792.s007.docx]

R packages used in the analyses of the manuscript

ape

readxl

cluster

dplyr

psych

car

MuMIn

ggplot2

scales
