# Supplementary material for: Degradation in landscape matrix has diverse impacts on diversity in protected areas
Source: PLoS One. 2017 Sep 26;12(9):e0184792. doi: 10.1371/journal.pone.0184792 (PMC5614538; doi:10.1371/journal.pone.0184792)
Supplement: S9 Text — (DOCX) [file pone.0184792.s009.docx]

Habitat categories in Finnish Point Counts

1. Spruce forest (spruce as dominating tree)

2. Pine forest (pine as dominating tree)

3. Deciduous forest (deciduous trees dominating)

4. Mixed forest (deciduous and coniferous trees equally abundant)

5. Deciduous shrub (< 5 m high)

6. Coniferous saplings (< 5 m high)

7. Clear-cut area

8. Pine swamp

9. Open mire

10. Waterside meadow

11. Agricultural field

12. Rural area (buildings, yards, gardens etc.)

13. Park

14. Urban area

15. Mountain birch forest

16. Treeless felltop

17. Other
